# NPAS3 variants in schizophrenia: a neuroimaging study

**DOI:** 10.1186/1471-2350-15-37

**Published:** 2014-03-27

**Authors:** Denise Bernier, Georgina Macintyre, Robert Bartha, Christopher C Hanstock, David McAllindon, Diane Cox, Scot Purdon, Katherine J Aitchison, Benjamin Rusak, Philip G Tibbo

**Affiliations:** 1 Department of Psychiatry, Dalhousie University, Halifax, Canada; 2 Department of Medical Genetics, University of Alberta, Edmonton, Canada; 3 Department of Diagnostic Radiology and Nuclear Medicine, Robarts Research Institute, University of Western Ontario, London, Canada; 4 Department of Biomedical Engineering, University of Alberta, Edmonton, Canada; 5 Department of Psychiatry, University of Alberta, Edmonton, Canada; 6 Departments of Psychiatry and Medical Genetics, University of Alberta, Edmonton, Canada; 7 Departments of Psychiatry, Psychology and Pharmacology, Dalhousie University, Halifax, Canada

**Keywords:** Relaxation time constants, ^1^H-MRS, *N*-acetylaspartate, Hippocampus, Temporal lobe, Psychosis, Genetics

## Abstract

**Background:**

This research is a one-site neuroimaging component of a two-site genetic study involving patients with schizophrenia at early and later stages of illness. Studies support a role for the neuronal *Per-Arnt-Sim 3* (*NPAS3*) gene in processes that are essential for normal brain development. Specific *NPAS3* variants have been observed at an increased frequency in schizophrenia. In humans, NPAS3 protein was detected in the hippocampus from the first trimester of gestation. In addition, NPAS3 protein levels were reduced in the dorsolateral prefrontal cortex of some patients with schizophrenia. *Npas3* knockout mice display behavioural, neuroanatomical and structural changes with associated severe reductions in neural precursor cell proliferation in the hippocampal dentate gyrus. This study will evaluate the hypothesis that the severe reductions in neural precursor cell proliferation in the dentate gyrus will be present to some degree in patients carrying schizophrenia-associated *NPAS3* variants and less so in other patients.

**Methods/Design:**

Patients enrolled in the larger genetic study (n = 150) will be invited to participate in this neuroimaging arm. The genetic data will be used to ensure a sample size of 45 participants in each genetic subgroup of patients (with and without *NPAS3* variants). In addition, we will recruit 60 healthy controls for acquisition of normative data. The following neuroimaging measures will be acquired from the medial temporal region: a) an index of the microcellular environment; b) a macro-structural volumetric measure of the hippocampus; and c) concentration levels of *N*-acetylaspartate, a marker of neuronal health.

**Discussion:**

This study will help to establish the contribution of the *NPAS3* gene and its variants to brain tissue abnormalities in schizophrenia. Given the genetic and phenotypic heterogeneity of the disorder and the large variation in outcomes, the identification of biological subgroups may in future support tailoring of treatment approaches in order to optimize recovery.

## Background

### Rationale

Psychotic disorders, including schizophrenia, are among the most devastating of all medical conditions in terms of personal suffering, disability, and costs to society. The personal suffering combined with the rising costs associated with this illness underscore the necessity of better understanding the biological underpinnings of psychotic disorders. This improved understanding should facilitate the development, implementation and evaluation of more effective and targeted treatment and rehabilitation programs.

With onset typically occurring during adolescence or early adulthood, schizophrenia is characterized by positive symptoms (delusions, hallucinations, disorganization of thought and behavior), negative symptoms (poverty of thought and affect, apathy and emotional withdrawal), in addition to cognitive symptoms (deficits in working memory and executive function). Even following treatment, significant residual symptoms may persist indefinitely, entailing a level of social and occupational disability. As a result of the early age of onset and the long term disability that can arise, the World Health Organization (WHO) proclaimed schizophrenia as ‘youth’s greatest disabler’. Despite increasing evidence for a host of neurobiological and genetic markers associated with schizophrenia, a full understanding of the biological underpinnings of this illness remains elusive [1]. A better understanding of how neurobiological, genetic and functional features of schizophrenia are linked together is essential in order to facilitate early identification of the illness, effective treatment, reduction of social and personal costs, and decreased disability.

### Schizophrenia candidate genes

Suggested candidate genes include, but are not limited to, neuregulin 1 (*NRG1)*, disrupted in schizophrenia-1 (*DISC1),* and catechol-1-methyltransferase (*COMT)*[2]. Despite strong support for the involvement of some genes, no defect in a single gene and no pattern of defects in a small set of genes have been identified as contributors to the majority of schizophrenia cases. Studies on European, African-American, Japanese and Chinese populations suggest that varied combinations of quite large numbers of genes likely contribute to the onset and development of schizophrenia, and that the specific gene combinations may differ with respect to ethnicity [3-5].

### Identification of a candidate gene for schizophrenia

Two research groups identified a Scottish family in which a mother and two daughters were affected with schizophrenia and carried a chromosome translocation, t(9;14)(q34:q13) [6,7]. Flow-sorted chromosomes from both mother and one available daughter were used to map a translocation breakpoint between the markers D14S730 and D14S70, a 683 kb interval at chromosome 14q13. Further molecular studies indicated that this breakpoint disrupted the neuronal *Per-Arnt-Sim 3* (*NPAS3*) gene, indicating a potential causative or contributory role to schizophrenia [6]. Nuclear localization of a green fluorescent (GFP)-labeled NPAS3 protein was observed, which was expected, as NPAS3 is a member of a family of transcription factors [8]. *Npas3* gene expression in the mouse is found mainly in the central nervous system, particularly in the neural tube, neuroepithelium and cortex [8,9].

Since the initial discovery, other groups have continued to explore the role of NPAS3 in nervous system development. During human embryogenesis, NPAS3 can be detected as early as the first trimester in numerous regions including the hippocampus and cerebellum [10]. MicroRNA control is characteristic of genes required for brain development, and NPAS3 is regulated post-natally by the microRNA miR-17 [11]. A search for genes controlled by NPAS3 has revealed pathways associated not only with neurogenesis, but also with glycolysis [12].

From an evolutionary perspective, the *NPAS3* gene was found to contain a ‘human accelerated region’, HAR21 [13]. Further studies revealed fourteen ‘human accelerated elements’ or HAEs associated with the gene, the largest cluster in the human genome [14]. These DNA elements are implicated in the development of human-specific traits, including brain evolution and function. Zebrafish studies indicate that many of the *NPAS3*-associated HAEs act as transcriptional enhancers of *NPAS3* expression during nervous system development, leading to the suggestion that they may influence spatio-temporal expression of NPAS3 in the human brain throughout development [14].

These studies provide further support for our hypothesis that NPAS3 influences brain structure and activity from the very earliest stages of human brain development.

### *NPAS3* variants in schizophrenia

*NPAS3* is comprised of twelve relatively short exons separated by large introns [6,7]. By full sequencing of all exons of the *NPAS3* coding region (sequenced individually), we identified two exons with differences from the published sequence. These two exons were subsequently sequenced in a cohort of 83 individuals with schizophrenia, compared to 83 controls [15].

Our case–control study identified significant changes in the *NPAS3* coding sequence with a positive association to schizophrenia. Three *NPAS3* variants occurred at a higher frequency in the patient group, and were in linkage disequilibrium in the vast majority of cases (rs12434716, p = 0.009; rs10141940 and rs10142034 both at p = 0.01). A variety of programs were used to predict the potential impact of the variants on *NPAS3* gene expression or protein function, including Sorting Intolerant from Tolerant (SIFT) to identify conserved amino acids function [16], Exon Splice Enhancer (ESE) Finder to locate putative motifs that regulate splicing [17,18], and NetPhos to identify potential phosphorylation motifs. The allele frequency of *NPAS3* single-nucleotide polymorphism (SNP) rs12434716 results in an amino acid change (p.Ala552Pro) and may influence the activity of NPAS3 protein. While the remaining SNPs are synonymous, some are located in exon splice enhancer motifs and may affect splicing. Thus, some of the SNPs may affect *NPAS3* function directly or be in linkage disequilibrium with the disease, causing change elsewhere in the gene [15].

Further support for NPAS3 variation in brain dysfunction came from a separate and concomitant case–control study using larger cohorts of individuals with schizophrenia (n = 386) or bipolar disorder (n = 368). Linkage-disequilibrium (LD)-based tag SNPs were examined across the *NPAS3* gene region, and both risk and protective interacting haplotypes were identified in four regions that differed from controls (n = 455) [19].

### The *Npas3* knockout mouse: a model for schizophrenia

Our hypothesis that NPAS3 variation may alter brain function and contribute to schizophrenia is supported by observations in *Npas3* deficient mice. The first report described the consequences of disrupting *Npas3* and the closely related *Npas1* gene, followed by two descriptions of the *Npas3* knockout mice that are particularly relevant to schizophrenia [9,20,21].

#### Behavioral abnormalities

A battery of behavioral and psychological tests was administered to the mice. A comparison of wild type mice (*Npas1*^+/+^*Npas3*^+/+^) with all possible genetic combinations revealed significant differences in relation to three specific genotypes: *Npas1*^−/−^*Npas3*^−/−^, *Npas1*^−/−^*Npas3*^+/−^ and *Npas1*^+/+^*Npas3*^−/−^. The knockout combinations showed a spectrum of behavioral and neurological abnormalities, implicating a role for defects in both genes in the development of schizophrenia. Behavioural abnormalities included diminished startle response, as measured by pre-pulse inhibition, gait defects, increased open-field activity, and impaired social recognition. These changes were more apparent in the *Npas3* null mice. An inability to prepare a nest or care for a litter and stereotypic darting behavior were observed exclusively in *Npas3*^*−/−*^ mice [20,21].

#### Structural and biochemical changes

In the adult mouse brain, *Npas1* and *Npas3* are expressed in inhibitory interneurons, which are essential to the correct processing of complex information received by the brain. A marked decrease in the expression of reelin was evident in inhibitory interneurons in *Npas1*^*+/−*^*Npas3*^*−/−*^ and *Npas1*^−/−^*Npas3*^−/−^ mice. Reelin is critical for the normal patterning of the embryonic brain that guides new neurons to specific locations in the appropriate layer of the developing cortex, and may have synaptic signalling functions in GABAergic interneurons [20]. Severe reductions in neural precursor cell proliferation in the hippocampal dentate gyrus were observed in *Npas3*^−/−^ mice, which correlated with a specific thinning of the granular layer of the dentate gyrus. The neurogenesis defect was reversible by electroconvulsive treatment [21,22]. Enlargement of ventricles was noted as well as altered foliation patterns in the cerebellum, and MRI analysis revealed changes in the midline crossing of the fibres of the corpus callosum [9].

The analogy between humans with *NPAS3*-related schizophrenia and mouse models of the illness might not be straightforward, however. These mice have both copies of the *Npas3* gene deleted, while the first family described has one defective gene, with the other presumed to be normal. In our case–control study, chromosome 14 is intact and the patients of interest usually displayed some specific variation in only one of the two alleles. Therefore, it is important to further assess the impact of this genetic variation in humans.

The larger genetic study (conducted at two different sites in Canada) is currently expanding on our earlier preliminary work examining *NPAS3* variants in schizophrenia. Participants are being recruited during early phase schizophrenia (first episode) as well as during chronic illness. The specific goals are: 1) to extend our case control study; 2) to carry out extensive evaluation of participants with schizophrenia; 3) to assess the association of *NPAS3* variation with clinical presentation and cognitive function; 4) to develop molecular assays to measure *NPAS3* variant function.

### Neuroimaging the impact of *NPAS3* variants

Based on evidence of abnormal temporal lobe development in mice with *Npas3* defects, our neuroimaging focus of interest in human brain will be the medial temporal region, which includes the hippocampal formation [21,22]. We will use several magnetic resonance imaging tools, including volumetrics, spectroscopy and measures of transverse relaxation time constants, in order to investigate the relationship between *NPAS3* genetic variation and human brain structure and function.

#### Transverse relaxation time constants

One neuroimaging measure that will be obtained relates to a microstructural feature of the medial temporal region. We will assess the transverse relaxation time constants of *N*-acetylaspartate (NAA), a neurochemical located intracellularly in mature neurons. *In vivo* concentration levels of NAA are slightly higher in white matter brain regions relative to gray matter regions [23]. Post-mortem studies have demonstrated that NAA is synthesized in neurons, transported into white matter cells and catabolized into aspartate and acetate in oligodendrocytes via aspartoacylase [24]. This neuroimaging index will provide an estimation of intracellular structural density in the brain region studied [25]. Transverse relaxation time constants are dependent on the number of molecule-microenvironment interactions: a greater number of interactions results in shorter relaxation times of the molecules of interest.

Declines in *in vivo* transverse relaxation time constants of NAA have been previously documented in relation to aging in healthy people [26], and also in relation to psychiatric illnesses like schizophrenia in frontal and parieto-occipital regions [25,27], as well as in a prefrontal region involving mainly white matter [28].

In terms of post-mortem studies, one study [29] found reduced packing density of hippocampal neurons in established schizophrenia, while other studies [30,31] reported no abnormality in that regard. Hippocampal cell size (a more subtle measure than cell packing density) has been reported to be reduced in schizophrenia [32].

We will also assess the transverse relaxation time constants of the regional water signal. This neuroimaging index will provide a measure of cell packing density involving both intracellular (cell density) and extracellular compartments (cell numbers) of the brain tissue of interest. There are few reports of *in vivo* studies assessing cell packing density of brain tissue in schizophrenia, and to our knowledge, no study has assessed potential abnormalities in these neuroimaging indices in relation to illness duration or genetic variants. These novel data will thus be useful in generating novel hypotheses in regards to the underpinnings of schizophrenia.

#### Hippocampal volume

A second neuroimaging index that will be obtained is at the level of macroscopic anatomy: a volumetric measure of the hippocampal formation. Structural differences in the brains of schizophrenia patients have been summarized in a meta-analysis that included 15 structural studies of schizophrenia [33]. The left temporal lobe (medial region) was the most consistent brain region observed with reduced volumes in schizophrenia (relative to healthy controls), although 30% of these studies did not report volumetric differences in this brain region. An analysis of the same measure linked to genetic features of patients, such as their *NPAS3* genotype, might help to clarify the basis for this variability.

A review of structural studies in schizophrenia [34] also pointed to the temporal and hippocampal regions, among others that might be altered in schizophrenia, with no overall evidence for progression of alterations over the course of illness or for sensitivity to medication status. The large variability and quality of methods used among studies was identified as a confounding factor leading to variable results. In the proposed study, manual editing and delineation of hippocampal volumes will be performed as the final step in hippocampal measurements in order to insure high validity. A previous report suggested that manual volume segmentation has greater validity relative to automated methods in accurately assessing volumes of small subcortical structures [35].

#### Proton magnetic resonance spectroscopy (^1^H-MRS)

A third neuroimaging measure will involve regional concentration levels of visible neurochemicals acquired with ^1^H-MRS. Although not a specific focus of this study, the ^1^H-MRS spectral profiles to be acquired for the calculation of transverse relaxation time constants of NAA will also provide information about regional concentration levels of NAA, choline compounds (Cho), and creatine plus phosphocreatine (tCr).

Previous ^1^H-MRS studies of schizophrenia assessing the temporal region have reported mixed and inconclusive results. Data compiled from studies with sample sizes greater than 20 patients with established schizophrenia, and that referenced neurochemicals to internal water, reported reduced regional levels of NAA [36-38], Cho and tCr [39] compared to controls. On the other hand, several other well-designed ^1^H-MRS studies of patients with schizophrenia have observed normal concentration levels in the temporal region for NAA [39-42], Cho and tCr [36,38,41,42]. In patients with first episode psychosis, normal concentration levels of all ^1^H-MRS visible neurochemicals have been reported in the temporal region [43,44]. In sum, despite some evidence for reduced concentration levels of NAA, Cho or tCr in the temporal region of patients with schizophrenia, there are also numerous negative reports from well-designed studies.

## Presentation of the hypotheses

This neuroimaging study will evaluate the hypothesis that the severe reductions in neural precursor cell proliferation in the hippocampal dentate gyrus observed in *Npas3*^−/−^ mice will also be present to some degree in those patients with schizophrenia displaying *NPAS3* genetic defects, and less so in other patients.

More specifically, we expect:

a) to replicate findings of reduced transverse relaxation time constants of NAA previously found in other brain regions [25,27], and of reduction in macroscopic structural volumetric measures previously found in the temporal region [33];

b) to observe greater levels of deviation from normal values in neuroimaging indices for the subgroup of patients displaying a defect in the *NPAS3* gene, relative to patients who don’t display this genetic variation (previously unstudied);

c) to observe a greater magnitude of abnormalities in neuroimaging indices measured at the micro-cellular level (transverse relaxation time constants) relative to the indices measured at the macro-anatomical level (previously unstudied);

d) to observe an association between degree of abnormality in brain measures and duration of illness, in patients displaying the genetic defect (previously unstudied).

## Testing the hypothesis

### A. The larger, two-site genetic study

We are currently completing a nationally funded genetic study at two different sites, in order to extend our previous case control study and continue the evaluation of the *NPAS3* gene in schizophrenia. The genetic study involves an extensive battery of clinical and cognitive assessments, along with genetic data collection (from saliva samples). Full ethics approval has been obtained for both sites and patients are currently being enrolled in the study, while a pool of DNA samples from matched volunteers with Caucasian origin will serve as control data.

At the specific site where the neuroimaging arm will take place (Halifax), 150 patients will be recruited over three years for the larger genetic study. About half of these patients will be at the early stages of the illness (<5 years since diagnosis) while the other patients will have chronic illness.

#### Statistical power analyses

In the previous case control study, we found significant differences between patients and healthy controls for some common *NPAS3* variants. For example, some variants occurred at a genotype frequency of ~30% (heterozygotes) in the schizophrenia population, compared with ~20% in healthy controls [15]. We also identified rare sequence variants, observed solely in the schizophrenia population, at a frequency of 1.2 to 3.6%. If we assume that these variants are disease-associated and occur at a similar frequency for all groups with schizophrenia, we would expect to find the more common *NPAS3* alleles in 45/150 (~7/120 homozygotes) in the cohort recruited at the location of the neuroimaging arm cohort, and detect rare *NPAS3* variants in 2 to 5 individuals.

### B. The one-site neuroimaging arm

#### Sample

Patients previously and currently enrolled in the genetic study (final n = 150) will be invited to participate in the neuroimaging arm. Ethics approval has been obtained to contact individuals who already participated in the genetic study and to use their genetic data in this neuroimaging arm. At time of writing, 60 patients have been enrolled in the genetic study with full attainment of sample size expected.

Patients from the genetic study will be categorized in two subgroups: those with (n = 45) or without (n = 105) *NPAS3* variants. We will use the available genetic data in order to ensure a minimal sample size of 45 in each genetic subgroup of patients in the neuroimaging protocol. In addition, the neuroimaging study will recruit 60 healthy controls, for compilation of normative data with respect to all relevant neuroimaging indices.

Healthy controls will be gender- and age-matched to patients; will have no lifetime diagnosis of psychiatric disorder; no first-degree relative with a diagnosis of psychosis; and taking no prescribed medications. Patients will be taking stable antipsychotic medications (these will be recorded and explored as potential confounding variables).

All participants will be Caucasian, will have no lifetime history of illicit drug usage of any type except for minimal experimentation (less than 10 times usage), and will have a history of alcohol usage generally in line with Canada’s Low-Risk Alcohol Drinking Guidelines [45].

#### Statistical power analyses

In terms of transverse relaxation time constants of NAA in schizophrenia relative to healthy controls, we calculated from previous data [27] that a sample size of 31 participants per group will provide adequate power to detect differences between independent groups, with a two-tailed test at an alpha level of .05 and power of .8.

In terms of volumetric measures of the hippocampus, no previous data exist with respect to subgrouping patients based on *NPAS3* genotypes. Given that the sample size planned for each group/subgroup (n = 45 each) is in line with most previous structural neuroimaging studies of schizophrenia [46], the subgrouping of patients on genetic grounds is likely to increase homogeneity of measurements within groups, and thus increase statistical power relative to previous structural studies.

Finally, with respect to ^1^H-MRS concentration levels quantified from spectra, a previous meta-analysis has calculated that across studies a minimal sample size of 39 patients in each group was required for adequate power to detect group differences in concentration levels of NAA [47].

#### Neuroimaging acquisitions

The anatomical images will be acquired with a 1.5 Tesla scanner using a 3D T_1_ weighted SPGR sequence involving 180 isometric slices acquired sagitally at a resolution of 1 mm with no interslice gap.

The brain volume of interest (VOI) is located in the left mid-temporal region, encompassing a large part of the hippocampal formation; its dimensions are 20 mm (R/L) by 28 mm (A/P) by 16 mm (S/I). Precise anatomical landmarks have been devised for reliable selection of the VOI.

Acquisitions of ^1^H-MRS neurochemical signals will be performed using a PRESS localization sequence with 80 acquisitions, time of repetition (TR) of 3 s and time of echo (TE) of 80, 120, 180, 350, and 550 ms. For the internal water signal, parameters are 4 acquisitions at TR of 10 s and TEs of 50, 60, 80, 120, 180, 350, 600, 800 and 1000 ms. Total duration of scanning will be approximately 75 min.

Relaxometry acquisitions for NAA start at a TE of 80 ms, in order to minimize the contribution from macromolecules into the estimation of neurochemicals (which would be present at shorter TE times). Relaxometry acquisitions for the water files start at a TE of 50 ms, in order to minimize the contribution from the myelin water signal into the estimations of CSF and tissue water relaxation times [48].

#### Neuroimaging offline analyses

The spectral profiles will be fitted using fitMAN [49] with a 3-peak manual fit of the three main signals: NAA, Cho, and tCr [50]. In order to inform future work, we will compile all the data acquired even when not part of the main research question. At 1.5 Tesla however, it is impossible to meaningfully interpret findings from the Cho signal, as the anabolic component (phosphocholine; PC) cannot be resolved separately from the catabolic component (glycerophosphocholine; GPC) of the total choline signal. On the other hand the tCr signal can provide very interesting information in the context of its decay at several different TEs. This signal contains two different components: phosphocreatine (PCr) and creatine (Cr), both involved in the energy equilibrium in brain cells [51]. Since PCr has much shorter T_2_ time constants relative to Cr [52], each individual component of the tCr signal can, with this design, be evaluated separately in terms of group differences.

From the NAA signal decay in amplitude over several TEs, the corresponding transverse relaxation time constants will be estimated as a curve fit of a mono-exponential decay [52]. Area under the water signal peak is calculated from the frequency domain at each TE using a MATLAB script. From the decrease in peak area as a function of TE, the corresponding T_2_ relaxation time constants are estimated as a curve fit of a two-component exponential decay using the Curve Fitting Tool in MATLAB [53]. The decay curve of water could potentially involve three components: CSF (long T_2_), tissue water (intermediate T_2_), and myelin water (very short T_2_) [48]. With this study design, signals from myelin water are mostly decayed at the echo times sampled; the very short myelin component is therefore not identified by the fitting algorithm.

Neurochemical concentration levels will be estimated at TE = 0 ms, using estimates of T_1_ and T_2_ time constants for the various tissue types and the internal water signal as a reference. Data will only be compiled in statistical analyses if the following quality criteria are met: uncertainty in the estimation of the fit (Cramer Rao lower bounds) lower than 20%, signal-to-noise ratio greater than 10, and no artifact present in the spectral profile. Tissue type segmentation of the VOI will be performed using the software packages FSL [54] and AFNI [55]. These procedures are automated with a customised script and are routinely used in our current ^1^H-MRS studies.

#### Segmentation of brain images

We will use the FSL module, FIRST, for an initial automated segmentation of subcortical structures, including hippocampus and amygdala. The image is first co-registered with the MNI 152 T_1_-weighted standard brain template at 1 mm resolution. This co-registration is done in two stages: first, using a 12 DOF affine registration and second, another 12 DOF affine registration optimized for subcortical structures by using an MNI-space subcortical mask. Segmentation is done using a library of shapes of the subcortical structures (based on 336 data sets manually segmented) to fit surface meshes of each structure to the registered image. Following the registration and segmentation, a boundary correction method is run to remove any overlap between structures in the native space. The results are stored in individual files for each structure as well as a single file that contains all the segmentations. The hippocampus volume will be determined from this file (FSL includes a utility to determine the volume of a segmented file). Total brain volumes will also be acquired for purposes of covariance with hippocampal volumes. Manual revision and editing of the automated segmented images will be performed in AFNI using an interactive 3D display. This manual editing procedure will insure optimal validity of volumetric measurements `[35]. Reliability of manually edited volumetric measures will be assessed between two raters and a concordance coefficient of at least 0.9 will be required.

### Planned statistical analyses

All statistical analyses will be performed with SPSS, and the threshold for significance will be set at .05, two-tailed. The Group factor will have 3 levels: patients with or without any of the three studied *NPAS3* genetic variants, and healthy controls. Potential Group differences in transverse relaxation time constants (in ms) of NAA and of regional tissue water will be assessed with a multivariate analysis of variance (MANOVA), followed by appropriate follow-up tests if the MANOVA is significant. In order to detect potential Group differences in regional concentration levels of the three visible ^1^H-MRS neurochemicals, we will also compute a MANOVA and appropriate follow-up tests if the MANOVA would be significant. Volumetric measures of the hippocampal formation will be contrasted between the three groups using an ANOVA, followed by post-hoc tests if the ANOVA would be significant.

Potential confounding variables could have an impact on neuroimaging measures. These variables include age, gender, stage of illness, medication status, as well as levels of psychotic symptoms, depression, and anxiety. Detailed information about these variables is collected as part of the current genetic study. Exploratory correlations will be computed between these variables and the neuroimaging measures in order to search for potential associations. In the potential case of an association between two relevant variables, appropriate covariance or regression analyses would be used.

## Implications of the hypothesis

To our knowledge, this is the first *in vivo* neuroimaging study investigating associations between *NPAS3* genetic variants and irregularities in the microscopic anatomy (transverse relaxation time constants), macroscopic anatomy (hippocampal volumes) and neurochemical profiles (^1^H-MRS) of the temporal region in schizophrenia. Irregularities have been reported in *Npas3* knockout mice and are hypothesized to also occur in individuals with schizophrenia. The implications of obtaining these pioneer data are to lead toward the discrimination of different subtypes of patients based on biological features, including genetic markers. Our hope is that in the near future, treatments will be tailored to specific biological subtypes in order to optimize outcomes.

### Ethics approval and financial support

Full ethics approval to conduct this neuroimaging study was received from the Capital Heath Research Ethics Board. Capital Health is a public provider of health care services in Halifax, Nova Scotia, Canada. Ethics approval was also obtained from the IWK Research Ethics Board, as the scanner is located in this hospital. The IWK Health Centre is a public provider of health care in Halifax. Each participant will be fully informed of the study prior to signing the consent form. Seed funding for this study was initially obtained from the Department of Psychiatry Research Fund at Dalhousie University. This neuroimaging study is currently financially supported by the Nova Scotia Health Research Foundation, Canada.

## Competing interests

PGT has served on advisory boards for Janssen, Roche, Sunovion and BMS, and has received speakers from Janssen, Roche and Sunovion. SP has received compensation for lectures and/or consultation from Lundbeck, Pfizer, Eli Lilly, Janssen, Novartis and AstraZeneca. BR serves as a paid consultant for the Institut de Recherches Internationales Servier. The other authors declare that they have no competing interests.

## Authors’ contributions

All authors have made intellectual contributions to the writing and editing of the full study proposal. DB made a substantial contribution to the conception and design of the neuroimaging arm of this proposal. GM made a substantial contribution to the conception and design of the genetic arm of this proposal. DM made a substantial contribution to all MR technical aspects of this proposal namely, pre-testing of MR data quality; computing of the decay curves and T_2_ relaxation time constants; and all aspects of compilation of MR data. RB provided his program for ^1^H-MRS offline analyses (fitMAN) and also provided technical support for the program in the context of 3-peak fits at 1.5 T, along with extensive teaching about offline analyses. CCH has designed the relaxometry parameters for acquisition and offline analyses; he also provided a custom-made program to assess the decay curves. RB and CCH made a substantial intellectual contribution to the writing of the ^1^H-MRS technical part of the proposal. KJA holds an Alberta Centennial Addiction and Mental Health Research Chair funded by the Government of Alberta. PGT provided help with recruitment and diagnosis of patients, and he supported the research as the Director of the NSEPP and as the Dr. Paul Janssen Chair in Psychotic Disorders. All authors have read and approved the final version of this study proposal.

## Pre-publication history

The pre-publication history for this paper can be accessed here:

http://www.biomedcentral.com/1471-2350/15/37/prepub

## References

[B1] Karlsgodt KH Jacobson SC Seal SC Fusar-Poli P The relationship of developmental changes in white matter to the onset of psychosis Curr Pharm Des 2012 15 422 433 10.2174/138161212799316073 22239573 PMC7130450

[B2] Ayalew M Le-Niculescu H Levey DF Jain N Changala B Patel SD Winiger E Breier A Shekhar A Amdur R Koller D Numberger JI Corvin A Geyer M Tsuang MT Salomon D Schork NJ Fanous AH O'Donovan MC Niculescu AB Convergent functional genomics of schizophrenia: from comprehensive understanding to genetic risk prediction Mol Psychiatry 2012 15 887 905 22584867 10.1038/mp.2012.37 PMC3427857

[B3] Allen NC Bagade S McQueen MB Ioannidis JP Kawoura FK Khouri MJ Tanzi RE Bertram L Systematic meta-analyses and field synopsis of genetic association studies in schizophrenia: the Sz Gene database Nat Genet 2008 15 827 834 10.1038/ng.171 18583979

[B4] Li M Zhang H Luo XJ Gao L Qi XB Gourraud PA Gourraud PA Su B Meta-analysis indicates that the European GWAS-identified risk SNP rs1344706 within ZNF804A is not associated with schizophrenia in Han Chinese population PLoS One 2013 15 e65780 10.1371/journal.pone.0065780 23776546 PMC3680487

[B5] Tsutsumi A Glatt SJ Kanazawa T Kawashige S Uenishi H Hokyo A Kaneko T Moritani M Kikuyama H Koh J Matsumura H Yoneda H The genetic validation of heterogeneity in schizophrenia Behav Brain Funct 2011 15 43 10.1186/1744-9081-7-43 21981786 PMC3198897

[B6] Kamnasaran D Muir WJ Ferguson-Smith MA Cox DW Disruption of the neuronal PAS3 gene in a family affected with schizophrenia J Med Genet 2003 15 325 332 10.1136/jmg.40.5.325 12746393 PMC1735455

[B7] Pickard BS Malloy MP Porteous DJ Blackwood DH Muir WJ Disruption of a brain transcription factor, NPAS3, is associated with schizophrenia and learning disability Am J Med Genet B Neuropsychiatr Genet 2005 15 26 32 10.1002/ajmg.b.30204 15924306

[B8] Kamnasaran D Chen CP Devriendt K Mehta L Cox DW Defining a holoprosencephaly locus on human chromosome 14q13 and characterization of potential candidate genes Genomics 2005 15 608 621 10.1016/j.ygeno.2005.01.010 15820313

[B9] Brunskill EW Ehrman LA Williams MT Klanke J Hammer D Schaefer TL Sah R end Dorn GW Potter SS Vorthees CV Abnormal neurodevelopment, neurosignaling and behaviour in Npas3-deficient mice Eur J Neurosci 2005 15 1265 1276 10.1111/j.1460-9568.2005.04291.x 16190882

[B10] Moreira F Kiehl T-R So K Ajeawung NF Honculada C Gould P Pieper RO Kamnasaran D NPAS3 demonstrates features of a tumor suppressive role in driving the progression of astrocytomas Am J Pathol 2011 15 462 476 10.1016/j.ajpath.2011.03.044 21703424 PMC3123785

[B11] Wong J Duncan CE Beveridge NJ Webster MJ Cairns MJ Weickert CS Expression of NPAS3 in the human cortex and evidence of its posttranscriptional regulation by miR-17 during development, with implications for schizophrenia Schizophr Bull 2013 15 396 406 10.1093/schbul/sbr177 22228753 PMC3576160

[B12] Sha L MacIntyre L Machell JA Kelly MP Porteous DJ Brandon NJ Muir WJ Blackwood DH Watson DG Clapcote SJ Pickard BS Transcriptional regulation of neurodevelopmental and metabolic pathways by NPAS3 Mol Psychiatry 2012 15 267 279 10.1038/mp.2011.73 21709683

[B13] Pollard KS Salama SR King B Kern AD Dreszer T Katzman S Siepel A Pedersen JS Bejerano G Baertsch R Rosenbloom KR Kent J Haussler D Forces shaping the fastest evolving regions in the human genome PLoS Genet 2006 15 e168 10.1371/journal.pgen.0020168 17040131 PMC1599772

[B14] Kamm GB Pisciottano F Kliger R Franchini LF The developmental brain gene NPAS3 contains the largest number of accelerated regulatory sequences in the human genome Mol Biol Evol 2013 15 1088 1102 10.1093/molbev/mst023 23408798 PMC3670734

[B15] Macintyre G Alford T Xiong L Rouleau GA Tibbo PG Cox DW Association of NPAS3 exonic variation with schizophrenia Schizophr Res 2010 15 143 149 10.1016/j.schres.2010.04.002 20466522

[B16] Saunders CT Baker D Evaluation of structural and evolutionary contributions to deleterious mutation prediction J Mol Biol 2002 15 891 901 10.1016/S0022-2836(02)00813-6 12270722

[B17] Cartegni L Krainer AR Disruption of an SF2/ASF-dependent exonic splicing enhancer in SMN2 causes spinal muscular atrophy in the absence of SMN1 Nat Genet 2002 15 377 384 10.1038/ng854 11925564

[B18] Cartegni L Wang J Zhu Z Zhang MQ Krainer AR ESEfinder: a web resource to identify exonic splicing enhancers Nucleic Acids Res 2003 15 3568 3571 10.1093/nar/gkg616 12824367 PMC169022

[B19] Pickard BS Christoforou A Thomson PA Fawkes A Evans KL Morris SW Porteous DJ Blackwood DH Muir WJ Interacting haplotypes at the NPAS3 locus alter risk of schizophrenia and bipolar disorder Molecular Psychiatry 2009 15 874 884 10.1038/mp.2008.24 18317462

[B20] Erbel-Sieler C Dudley C Zhou Y Wu X Estill SJ Han T Diaz-Arrastia R Brunskill EW Potter SS McKnight SL Behavioral and regulatory abnormalities in mice deficient in the NPAS1 and NPAS3 transcription factors Proc Natl Acad Sci U S A 2004 15 13648 13653 10.1073/pnas.0405310101 15347806 PMC518807

[B21] Pieper AA Wu X Han TW Estill SJ Dang Q Wu LC Reece-Fincanon S Dudley CA Richardson JA Brat DJ McKnight SL The neuronal PAS domain protein 3 transcription factor controls FGF-mediated adult hippocampal neurogenesis in mice Proc Natl Acad Sci U S A 2005 15 14052 14057 10.1073/pnas.0506713102 16172381 PMC1216832

[B22] Pieper AA Xie S Capota E Estill SJ Zhong J Long JM Becker GL Huntington P Goldman SE Shen CH Capota M Britt JK Kotti T Ure K Brat DJ Williams NS MacMillan KS Naidoo J Melito L Hsieh J De Brabander J Ready JM McKnight SL Discovery of a proneurogenic, neuroprotective chemical Cell 2010 15 39 51 10.1016/j.cell.2010.06.018 20603013 PMC2930815

[B23] Hetherington HP Mason GF Pan JW Ponder SL Vaughan JT Twieg DB Pohost GM Evaluation of cerebral gray and white matter metabolite differences by spectroscopic imaging at 4.1 T Magn Reson Med 1994 15 565 571 10.1002/mrm.1910320504 7808257

[B24] Moffet JR Ross B Arun P Madhavarao CN Namboodiri AMA *N*-acetylaspartate in the CNS: From neurodiagnosis to neurobiology Prog Neurobiol 2007 15 89 131 10.1016/j.pneurobio.2006.12.003 17275978 PMC1919520

[B25] Öngür D Prescot AP Jensen JE Rouse ED Cohen BM Renshaw PF Olson DP T2 relaxation time abnormalities in bipolar disorder and schizophrenia Magn Res Med 2010 15 1 8 10.1002/mrm.22148 19918902

[B26] Kirov II Fleysher L Fleysher R Patil V Liu S Gonen O Age dependence of regional proton metabolites *T*_*2*_ relaxation times in the human brain at 3 T Magn Reson Med 2008 15 790 795 10.1002/mrm.21715 18816831 PMC2631566

[B27] Tunc-Skarka N Weber-Fahr W Hoerst M Meyer-Lindenberg A Zink M Ende G MR spectroscopic evaluation of N-acetylaspartate’s T2 relaxation time and concentration corroborates white matter abnormalities in schizophrenia NeuroImage 2009 15 525 531 10.1016/j.neuroimage.2009.06.061 19573608

[B28] Du F Cooper A Cohen BM Renshaw PF Öngür D Water and metabolite transverse T2 relaxation time abnormalities in the white matter in schizophrenia Schizophr Res 2012 15 241 245 10.1016/j.schres.2012.01.026 22356802 PMC3351533

[B29] Jeste DV Lohr JB Hippocampal pathologic findings in schizophrenia. A morphometric study Arch Gen Psychiatry 1989 15 1019 1024 10.1001/archpsyc.1989.01810110061009 2818139

[B30] Benes FM Sorensen I Bird ED Reduced neuronal size in posterior hippocampus of schizophrenic patients Schizophr Bull 1991 15 597 608 10.1093/schbul/17.4.597 1805353

[B31] Falkai P Bogerts B Rozumec M Limbic pathology in schizophrenia Biol Psychiatry 1988 15 515 521 10.1016/0006-3223(88)90162-X 3167140

[B32] Heckers S Konradi C Hippocampal neurons in schizophrenia J Neural Transm 2002 15 891 905 10.1007/s007020200073 12111476 PMC4205576

[B33] Honea R Crow TJ Passingham D Mackay CE Regional deficits in brain volume in schizophrenia: a meta-analysis of voxel-based morphomotry studies Am J Psychiatry 2005 15 2233 2245 10.1176/appi.ajp.162.12.2233 16330585

[B34] Shepherd AM Laurens KR Matheson SL Carr VJ Green MJ Systematic meta-review and quality assessment of the structural brain alterations in schizophrenia Neurosci Biobehav Rev 2012 15 1342 1356 10.1016/j.neubiorev.2011.12.015 22244985

[B35] Tae WS Kim SS Lee KU Nam EC Kim KW Validation of hippocampal volumes measured using a manual method and two automated methods (FreeSurfer and IBASPM) in chronic major depressive disorder Neuroradiology 2008 15 569 581 10.1007/s00234-008-0383-9 18414838

[B36] Chang L Friedman J Ernst T Zhong K Tsopelas ND Davis K Brain metabolite abnormalities in the white matter of elderly schizophrenic subjects: implication for glial dysfunction Biol Psychiatry 2007 15 1396 1404 10.1016/j.biopsych.2007.05.025 17693392 PMC2222890

[B37] Klär AA Ballmaier M Leopold K Häke I Schaefer M Brühl R Schubert F Gallinat J Interaction of hippocampal volume and N-acetylaspartate concentration deficits in schizophrenia: a combined MRI and 1H-MRS study NeuroImage 2010 15 51 57 10.1016/j.neuroimage.2010.06.006 20541020

[B38] Tang CY Friedman J Shungu D Chang L Ernst T Stewart D Hajianpour A Carpenter D Ng J Mao X Hof PR Buchsbaum MS Davis K Gorman JM Correlations between Diffusion Tensor Imaging (DTI) and Magnetic Resonance Spectroscopy (^1^H-MRS) in schizophrenic patients and normal controls BMC Psychiatry 2007 15 25 10.1186/1471-244X-7-25 17578565 PMC1929081

[B39] Maier M Ron MA Hippocampal age-related changes in schizophrenia: a proton magnetic resonance spectroscopy study Schizophr Res 1996 15 5 17 10.1016/0920-9964(96)00044-8 8908686

[B40] Szulc A Galiñska B Tarasów E Kubas B Dzienis W Konarzewska B Poplawska R Tornczak AA Czernikiewicz A Walecki J N-acetylaspartate (NAA) levels in selected areas of the brain in patients with chronic schizophrenia treated with typical and atypical neuroleptics: a proton magnetic resonance spectroscopy (^1^H-MRS) study Med Sci Monit 2007 15 17 22 17507880

[B41] Van Elst LT Valerius G Büchert M Thiel T Rüsch N Bubl E Hennig J Ebert D Olbrich HM Increased prefrontal and hippocampal glutamate concentration in schizophrenia: evidence from a magnetic resonance spectroscopy study Biol Psychiatry 2005 15 724 730 10.1016/j.biopsych.2005.04.041 16018980

[B42] Venkatraman TN Hamer RM Perkins DO Song AW Lieberman JA Steen RG Single-voxel ^1^H PRESS at 4.0 T: precision and variability of measurements in anterior cingulate and hippocampus NMR Biomed 2006 15 484 491 10.1002/nbm.1055 16763968

[B43] Galiñska B Szulc A Tarasów E Kubas B Dzienis W Czernikiewick A Walecki J Duration of untreated psychosis and proton magnetic resonance spectroscopy (^1^H-MRS) findings in first-episode schizophrenia Med Sci Monit 2009 15 CR82 CR88 19179972

[B44] Wood SJ Berger GE Wellard RM Proffitt T McConchie M Velakoulis D McGorry PD Pantelis C A ^1^H-MRS investigation of the medial temporal lobe in antipsychotic-naïve and early-treated first episode psychosis Schizophr Res 2008 15 163 170 18456460 10.1016/j.schres.2008.03.012

[B45] Canadian Centre on Substance Abuse Canada’s Low-Risk Alcohol Drinking Guidelines 2013 http://www.ccsa.ca/Eng/topics/alcohol/drinking-guidelines/Pages/default.aspx

[B46] Bora E Fornito A Radua J Walterfang M Seal M Wood SJ Yücel M Velakoulis D Pantelis C Neuroanatomical abnormalities in schizophrenia: a multimodal voxelwise meta-analysis and meta-regression analysis Schizophr Res 2011 15 46 57 10.1016/j.schres.2010.12.020 21300524

[B47] Steen RG Hamer RM Lieberman JA Measurement of brain metabolites by ^1^H magnetic resonance spectroscopy in patients with schizophrenia: a systematic review and meta-analysis Neuropsychopharmacology 2005 15 1949 1962 10.1038/sj.npp.1300850 16123764

[B48] MacKay A Laule C Vavasour I Bjarnason T Kolind S Mädler B Insights into brain microstructure from the T_2_ distribution Magn Res Imaging 2006 15 515 525 10.1016/j.mri.2005.12.037 16677958

[B49] Bartha R Drost DJ Williamson PC Factors affecting the quantification of short echo in-vivo 1H MR spectra: prior knowledge, peak elimination, and filtering NMR Biomed 1999 15 205 216 10.1002/(SICI)1099-1492(199906)12:4<205::AID-NBM558>3.0.CO;2-1 10421912

[B50] Bernier D Bartha R Devarajan S MacMaster FP Schmidt MH Rusak B Effects of overnight sleep restriction on brain chemistry and mood in women with unipolar depression and healthy controls J Psychiatry Neurosci 2009 15 352 360 19721845 PMC2732741

[B51] Tibbo P Bernier D Hanstock CC Seres P Lakusta B Purdon SE 3-T proton magnetic spectroscopy in unmedicated first episode psychosis: a focus on creatine Magn Reson Med 2013 15 613 620 10.1002/mrm.24291 22511463

[B52] Ke Y Cohen BM Lowen S Hirashima L Nassar L Renshaw PF Biexponential transverse relaxation (T_2_) of the proton MRS creatine resonance in human brain Magn Res Med 2002 15 232 238 10.1002/mrm.10063 11810665

[B53] Gilat A MATLAB: An introduction with Applications 2004 2 Mississauga: John Wiley & Sons

[B54] Woolrich MW Jbabdi S Patenaude B Chappell M Makni S Behrens T Beckmann C Jenkinson M Smith SM Bayesian analysis of neuroimaging data in FSL NeuroImage 2009 15 S173 S186 10.1016/j.neuroimage.2008.10.055 19059349

[B55] Cox RW AFNI: software for analysis and visualization of functional magnetic resonance neuroimages Comput Biomed Res 1996 15 162 173 10.1006/cbmr.1996.0014 8812068

